# Navigating the Frontier: Emerging Techniques for Detecting Microvascular Complications in Type 2 Diabetes Mellitus: A Comprehensive Review

**DOI:** 10.7759/cureus.53279

**Published:** 2024-01-31

**Authors:** Sarang S Raut, Sourya Acharya, Vinit Deolikar, Satish Mahajan

**Affiliations:** 1 General Medicine, Jawaharlal Nehru Medical College, Datta Meghe Institute of Higher Education and Research, Wardha, IND; 2 General Medicine, Jawaharlal Nehru Medical College, Datta Meghe Institute of Higher Education & Research, Wardha, IND

**Keywords:** personalized medicine, precision diagnostics, early detection, emerging techniques, microvascular complications, type 2 diabetes mellitus

## Abstract

This review comprehensively explores emerging techniques for detecting microvascular complications in Type 2 Diabetes Mellitus (T2DM), addressing the critical need for advancements in early detection and management. As T2DM continues to rise globally, microvascular complications, including retinopathy, nephropathy, and neuropathy, contribute significantly to the morbidity and mortality associated with the condition. The review synthesizes key findings, revealing various emerging technologies, from advanced imaging modalities to genomic and proteomic approaches. It underscores the potential for personalized medicine, emphasizing the importance of tailoring diagnostic strategies to individual patient profiles. Challenges, including the lack of standardized criteria and issues related to patient adherence, highlight the necessity for collaborative efforts. The conclusion issues a call to action, advocating for enhanced collaboration, increased research investment, patient empowerment through education, and seamless integration of emerging diagnostic techniques into routine clinical care. The review envisions a transformative shift in detecting and managing microvascular complications in T2DM, ultimately improving patient outcomes and contributing to a healthier future for individuals affected by this prevalent metabolic disorder.

## Introduction and background

Type 2 Diabetes Mellitus (T2DM) is a global health challenge characterized by insulin resistance and impaired glucose metabolism. This metabolic disorder not only affects millions worldwide but also carries a significant burden due to its associated complications. Among these complications, microvascular complications play a pivotal role, contributing substantially to morbidity and mortality in individuals with T2DM [1]. T2DM is a chronic condition marked by the body's inability to use insulin, leading to elevated blood glucose levels effectively. It is often associated with lifestyle factors such as sedentary behavior, unhealthy dietary habits, and genetic predisposition. As the prevalence of T2DM continues to rise globally, understanding its complications becomes imperative for effective management and improved patient outcomes [2].

Microvascular complications are a significant issue for patients with T2DM. These complications can have a substantial impact on morbidity and mortality. The prevalence of microvascular complications in newly diagnosed T2DM patients varies depending on the study and location. Here are some key findings from the search results: In a study of newly diagnosed T2DM patients, the prevalence of microvascular complications was 12% (IQR: 6%-15%) [3]. In another study conducted in Tianjin, China, the prevalence of microvascular complications was 34.5% [4]. A study conducted in the Middle East and Africa cohort found the overall crude prevalence of microvascular complications to be 17.7% [5]. Several factors have been identified as risk factors for microvascular complications in T2DM patients. These include longer diabetes duration, the presence of hypertension and dyslipidemia, and insulin use [4]. Early detection and intervention can help reduce complications and improve patient outcomes. However, more research is needed to confirm the causal relationship between these risk factors and microvascular complications [4].

This comprehensive review explores emerging techniques for detecting microvascular complications in individuals with T2DM. By synthesizing existing knowledge and presenting recent advancements, the review thoroughly explains the evolving landscape in diagnostics for microvascular complications. Early detection of microvascular complications is paramount in the management of T2DM. Timely identification allows for prompt intervention, reducing the progression of complications and mitigating their impact on patients' lives. Through this review, we aim to underscore early detection's critical role in shaping clinical outcomes, emphasizing the potential for improved prognosis and enhanced overall well-being for individuals living with T2DM.

## Review

Microvascular complications in type 2 diabetes mellitus

Overview of Microvascular Complications

Retinopathy: Retinopathy is a microvascular complication that affects small blood vessels in the eye's retina. It is a common complication of T2DM and can lead to vision loss if left untreated. In a study of newly diagnosed T2DM patients, the prevalence of retinopathy was 31.4% [6]. In another study conducted in Tianjin, China, the prevalence of retinopathy was 22.5% [4]. Diabetic retinopathy remains the most common cause of blindness in working-age adults in the developed world [7]. The pathophysiology of retinopathy is complex and involves multiple mechanisms, including hyperglycemia, oxidative stress, inflammation, and vascular dysfunction [7]. Early detection and intervention are crucial in preventing vision loss. Screening for retinopathy should be performed annually in T2DM patients, starting at the time of diagnosis [7]. Treatment options include laser therapy, intravitreal injections, and vitrectomy [7]. Tight glycemic control, blood pressure, and lipid management are also crucial in preventing and managing retinopathy [7].

Nephropathy: Nephropathy is another microvascular complication of T2DM that affects the kidneys. It can lead to severe renal disease and is a significant cause of morbidity and mortality in T2DM patients. In a study of newly diagnosed T2DM patients, the prevalence of nephropathy was found to be 56.2% [6]. In another study conducted in Tianjin, China, the prevalence of nephropathy was 20.5% [4]. Diabetic nephropathy is the leading cause of end-stage renal disease in developed countries [8]. The nephropathy pathophysiology is complex and involves various factors, such as hyperglycemia, oxidative stress, inflammation, and vascular dysfunction [8]. Early detection and intervention are crucial in preventing and managing nephropathy. Screening for nephropathy should be performed annually in T2DM patients, starting at the time of diagnosis [8]. Treatment options include lifestyle modifications, medication, and, in severe cases, dialysis or kidney transplantation [8]. Tight glycemic control, blood pressure, and lipid management are essential in preventing and managing nephropathy [8].

Neuropathy: Neuropathy is a common microvascular complication in patients with T2DM, affecting the peripheral nerves. The prevalence of neuropathy in newly diagnosed T2DM patients has been reported to be as high as 68.5% in some studies [6]. Diabetic neuropathy can lead to significant morbidity and mortality, causing neurological dysfunction in various organ systems, such as the cardiovascular and gastrointestinal systems [9]. The injurious effects of hyperglycemia are separated into macrovascular and microvascular complications, with diabetic neuropathy being a significant microvascular complication [9]. Management of diabetic neuropathy focuses on symptom control, and close monitoring for disease progression is essential to prevent long-term complications [9].

The link between Microvascular Complications and T2DM

The link between microvascular complications and T2DM is well established. Microvascular complications, such as retinopathy, neuropathy, and nephropathy, are common in patients with T2DM and can have a significant impact on morbidity and mortality. A study of newly diagnosed T2DM patients found that the prevalence of microvascular complications was estimated to be 12% (IQR: 6%-15%) [3]. Another study reported that in newly diagnosed T2DM patients, the prevalence of neuropathy, nephropathy, and retinopathy was 68.5%, 56.2%, and 31.4%, respectively [6]. These complications are a significant cause of morbidity and mortality in T2DM patients [3]. Microvascular complications are associated with a longer duration of diabetes and higher levels of glycated hemoglobin (HbA1c) [10]. Therefore, early detection and management of microvascular complications are crucial in the care of patients with T2DM.

Impact on Patient Health and Quality of Life

The impact of microvascular complications on the quality of life (QOL) of patients with T2DM is significant. Studies have shown that both microvascular and macrovascular complications of T2DM can worsen the quality of life of affected individuals. For instance, the development of macrovascular complications and neuropathy has been associated with decreases in the quality of life [11]. These complications can cause symptoms such as angina, claudication, dyspnea, and weakness, which can markedly impair patients' functional capacity and increase the risk for depression, thereby worsening their quality of life [11]. Additionally, pain/discomfort, mobility, and depression/anxiety have been identified as the main problems affecting the quality of life of diabetic patients [12]. Therefore, the effective management of microvascular complications is crucial for patients' physical health and overall quality of life.

Traditional methods of detecting microvascular complications

Routine Clinical Assessments

Traditional approaches to identifying microvascular complications in individuals with T2DM encompass routine clinical assessments and screenings. These methodologies are pivotal for the timely recognition and subsequent management of complications. For the detection of retinopathy, a prevalent cause of blindness in working-age adults with diabetes, it is recommended that T2DM patients undergo regular retinal screenings [7,8]. Screening involves the examination of urine for microalbumin and other indicators of kidney function to identify nephropathy. Aggressive early intervention for microalbuminuria has demonstrated efficacy in reducing the risk of developing nephropathy [7]. Neuropathy, which may manifest in diverse ways and prove challenging to address, is typically screened through methods like monofilament testing to evaluate the loss of protective sensation in the feet [7,8]. Undetected and untreated microvascular complications can significantly diminish the quality of life for individuals with T2DM. Therefore, routine screening and early detection are imperative to forestall or delay the onset of these complications, ultimately leading to improved patient outcomes [8,13]. The conventional methods employed for detecting microvascular complications play a vital role in the holistic management of T2DM, with the overarching goal of reducing the disease burden and enhancing the quality of life for affected individuals.

Biomarkers and Blood Tests

Traditional methods for identifying microvascular complications in T2DM involve using biomarkers and blood tests, facilitating early detection and effective management of complications. Blood-based biomarkers with potential applications in managing coronary microvascular disease have been identified, presenting opportunities for early detection and intervention [14]. Likewise, ongoing research aims to pinpoint biomarkers for vision-threatening diabetic retinopathy, potentially serving as future screening tools [15]. Advancements in technology have led to the development of wearable glucose monitors with heightened sensitivity to low glucose concentrations. These innovations offer non-invasive screening and monitoring options, contributing to early detection and management of microvascular complications [16].

However, the introduction of potential biomarkers into clinical practice requires thorough validation. This necessitates the design of appropriate clinical assays to ensure reliable results across significant patient samples [16]. Using biomarkers and blood tests in traditional methods plays a crucial role in the early identification and management of microvascular complications, ultimately enhancing the quality of life for individuals with T2DM. Further research and validation efforts are imperative to refine these diagnostic and screening capabilities. Exploring specific biomarkers, the neutrophil-lymphocyte ratio (NLR) emerges as a promising candidate. Studies suggest that an elevated NLR may indicate underlying impaired glucose metabolism and serve as a marker for diabetic control level alongside HbA1c in type 2 diabetes. Its association with impaired glycemic control and its potential as a marker for systemic inflammation and various complications, including diabetic nephropathy, underscores its significance. While promising, further research is needed to establish the clinical utility of NLR entirely [17].

Other biomarkers, such as sialic acid, quantitative imaging biomarkers, and genomics, have been considered for evaluating complications in T2DM, particularly those related to angiopathy and cardiovascular concerns [18]. Pentosidine studied as a potential biomarker for microvascular complications in type 2 diabetic patients, shows promise. Research indicates that elevated serum pentosidine levels are significantly associated with diabetic retinopathy and nephropathy, making it a potential biomarker for these microvascular complications. These findings suggest pentosidine's potential value in assessing the risk and presence of microvascular complications in individuals with type 2 diabetes [19].

Imaging Techniques

Fundus photography for retinopathy: Fundus photography is a widely used imaging technique for screening and detecting diabetic retinopathy (DR). It involves capturing high-quality retina images to identify any signs of retinopathy. However, traditional fundus photography has limitations, such as the inability to image the peripheral retina where DR lesions may be missed, and being a 2-dimensional technique, it can be challenging to assess for diabetic macular edema (DME) [20]. Newer retinal imaging technologies, such as ultra-widefield (UWF) fundus imaging and OCT, have been developed to address these limitations. UWF fundus imaging uses laser light and confocal principles to capture a more comprehensive view of the retina, including the periphery; it can be beneficial in detecting DR lesions that may be missed by traditional fundus photography [20]. On the other hand, OCT provides cross-sectional images of the retina, allowing for the detailed assessment of the macula and the detection of DME [21]. In addition to these advancements, selfie fundus imaging, where patients take photos of their retinas, has shown promise as a screening tool for DR. This approach can be beneficial in situations such as the ongoing COVID-19 pandemic, where traditional in-person screenings may be challenging [22]. While traditional fundus photography remains a valuable tool for DR screening, newer imaging technologies such as UWF fundus imaging and OCT, along with innovative approaches like selfie fundus imaging, are enhancing the capabilities for early detection and monitoring of diabetic retinopathy.

Renal imaging for nephropathy: Traditional methods of detecting microvascular complications, such as nephropathy in patients with T2DM, include various imaging techniques. These techniques help in the early identification and management of complications. Ultrasound is frequently used to measure kidney size, search for the presence of renal masses or cysts, detect kidney stones, and determine whether there is urinary obstruction [23]. Doppler imaging can be added to examine flow in the renal arteries and veins or measure blood flow velocity [23]. CT and MRI are also frequently used for renal imaging. However, potentially toxic contrast media limits their application to patients with an estimated glomerular filtration rate (eGFR) below 30 mL per minute [23]. Renal scintigraphy uses small amounts of radioactive material called radiopharmaceuticals, a special camera, and a computer to evaluate kidney function and anatomy and determine whether the kidneys are working correctly [24]. These traditional methods of detecting microvascular complications in T2DM patients involve various imaging techniques. These methods help in early identification and management of complications, improving the quality of life for affected individuals. However, more research is needed to identify the most sensitive and specific imaging techniques for the early detection and monitoring of microvascular complications in T2DM patients.

Nerve conduction studies (NCS) for neuropathy: NCS is a common diagnostic test used to evaluate peripheral neuropathy, a microvascular complication associated with T2DM. These studies help assess the function of peripheral nerves and can aid in diagnosing nerve damage. NCS is used to detect the presence and extent of peripheral nerve damage and to differentiate between various types of neuropathies, such as demyelinating and axonal neuropathies [25,26]. During the test, electrodes are placed on the skin along a nerve pathway, and the nerve is stimulated with a mild electrical impulse. The speed and strength of the nerve's response are then measured. This helps evaluate the conduction of electrical impulses along the nerves [25]. NCS may be indicated for patients with symptoms of peripheral neuropathy, such as tingling, numbness, weakness, or pain in the extremities. The test can help healthcare providers determine neuropathy's cause, severity, and prognosis [25]. The test results can provide information on the type and extent of nerve damage, which can be valuable for diagnosing and monitoring peripheral neuropathies. For example, demyelinating injuries may be characterized by prolonged distal slowed conduction velocity and prolonged F-wave latency [26]. Nerve conduction studies are a valuable tool for evaluating peripheral neuropathy, a common microvascular complication in patients with T2DM. These studies aid in diagnosing and differentiating various types of neuropathies, thus contributing to the comprehensive management of T2DM and its associated complications.

Limitations of Traditional Methods

The limitations of traditional methods for detecting microvascular complications in T2DM are evident in the current literature. While these methods, such as cardiovascular imaging, retinal screening, and nerve conduction studies, are valuable, they have certain constraints. For instance, the identification of microvascular disease, particularly in cardiovascular imaging, needs to be established compared to the detection of epicardial ischemic disease [27]. Additionally, the prevalence of microvascular complications in newly diagnosed T2DM patients, even with poor glycemic control, highlights the need for more robust and early detection methods [6]. Furthermore, the challenges in screening and detecting microvascular complications, such as retinopathy, nephropathy, and neuropathy, emphasize the importance of nationwide screening and robust screening methods to identify these complications early [7]. Therefore, while traditional methods play a crucial role, there is a need for more advanced and robust screening and detection techniques to address the limitations and improve the early identification of microvascular complications in T2DM patients.

Emerging techniques for detection

Advanced Imaging Technologies

Optical coherence tomography (OCT) for retinopathy: OCT is an emerging technology for the detection and monitoring of diabetic retinopathy (DR), a microvascular complication associated with T2DM. OCT is a non-invasive imaging technique that uses light waves to capture high-resolution cross-sectional images of the retina [28]. OCT provides detailed images of the retina, allowing for the detection of subtle changes in retinal thickness and morphology that may indicate the presence of DR. OCT can also detect diabetic macular edema (DME), a common complication of DR [29]. OCT is used in clinical practice to diagnose and monitor DR and DME. It is also used to evaluate the efficacy of treatments for these conditions [30]. Advances in OCT technology, such as ultra-high-resolution OCT and OCT angiography, are improving the capabilities of OCT for detecting and monitoring DR and DME [29]. While OCT is a valuable tool for detecting and monitoring DR and DME, it has certain limitations. For example, OCT cannot detect early-stage DR lesions that are not yet visible on fundus photography [29]. Additionally, OCT is only widely available in some clinical settings, and its cost may limit its use in some populations [29]. OCT is an emerging technology for detecting and monitoring DR and DME, two microvascular complications associated with T2DM. While OCT has certain limitations, its high-resolution imaging capabilities and clinical applications make it a valuable tool for comprehensively managing T2DM and its associated complications.

Magnetic resonance imaging (MRI) for nephropathy: Recent advances in MRI have shown promise in the evaluation of diabetic kidney disease (DKD), a common microvascular complication of T2DM. These advances include multiparametric MRI, which allows for collecting multiple quantitative measures to assess kidney morphology, tissue microstructure, oxygenation, kidney blood flow, and perfusion in a single scan session [31]. Additionally, diffusion-weighted imaging (DWI-MRI) has emerged as a promising method for assessing renal microstructure, particularly fibrosis, in DKD [32]. While traditional imaging methods, such as ultrasound, CT, and MRI, have been used to assess kidney size, density, and the presence of renal masses or cysts, recent advances in MRI techniques offer the potential for a more comprehensive and non-invasive evaluation of DKD. These advancements enable the assessment of renal volume, function, metabolism, perfusion, oxygenation, and microstructural alterations without the need for exogenous contrast media [33]. The use of multiparametric MRI and DWI-MRI in evaluating DKD represents a significant advancement in renal imaging, offering the potential for a more detailed and non-invasive assessment of kidney structure, function, and pathology in patients with T2DM. These emerging techniques promise to improve the early detection and monitoring of DKD, ultimately contributing to better patient outcomes.

Nerve fiber density measurements for neuropathy: MRI has emerged as a promising technique for assessing peripheral nerve fiber density, which can help diagnose neuropathy, a microvascular complication associated with T2DM. MRI has been shown to provide rich image contrast, high resolution, and more quantitative features that could be viable biomarkers for peripheral nerve pathology [34]. However, MRI has yet to be utilized in human peripheral nervous system (PNS) studies in vivo due to the small size of nerve structures and the lack of normative data for MRI in the PNS [34]. Recent advances in MRI technology, such as the increased availability of 3 Tesla (T) and 7T systems, have enabled MRI to study peripheral nerve disease. At higher field strength, MRI can offer fascicular level resolution in larger peripheral nerves [35]. Peripheral nerve MRI has been used to visualize morphological changes in the nerves, and the combination of diffusion-weighted imaging (DWI) and diffusion tensor imaging (DTI) could provide enough sensitivity to reveal pathological changes [34]. Advanced imaging techniques such as MRI, particularly with higher field strengths, offer promising prospects for detecting and assessing peripheral nerve fiber density in patients with neuropathy and T2DM. These emerging techniques can improve neuropathy's early diagnosis and management, ultimately improving patient outcomes.

Genomic and Proteomic Approaches

Genomic and proteomic methodologies represent innovative approaches for the early detection of microvascular complications in T2DM, providing a more comprehensive understanding of the molecular mechanisms involved and aiding in early diagnosis and treatment. Proteomics involves the extensive study of the structure and function of proteins within complex biological systems, offering insights into the molecular mechanisms underlying diseases, including T2DM [36]. High-throughput proteomics techniques, such as mass spectrometry and liquid chromatography-mass spectrometry, have significantly advanced the identification and quantification of proteins in biological samples [37].

On the genomic front, approaches like genome-wide association studies can assist in identifying genetic risk factors associated with T2DM and its microvascular complications [38]. Integrating genomics with other "omics," including transcriptomics and metabolomics, enhances our ability to comprehend the intricate nature of T2DM and microvascular complications [38]. Despite the rapid progress in proteomic technology, certain challenges persist in detecting, identifying, and quantifying proteins in biological samples. These challenges arise from the complexity of biological structures and the extensive data processing and analysis required [36]. Genomic and proteomic approaches hold significant promise for detecting and managing microvascular complications in T2DM patients. These emerging techniques contribute to an enhanced understanding of the molecular mechanisms underlying these complications, ultimately leading to improved patient outcomes. However, ongoing efforts are essential to address challenges and further refine these methodologies for effective application in clinical settings.

Point-of-Care Testing

Point-of-care testing (POCT) is rapidly evolving, with emerging technologies enhancing its effectiveness and applications. These advancements include the utilization of contemporary technologies such as the Internet of Things (IoT), artificial intelligence (AI), and machine learning to increase the accuracy and effectiveness of POCT devices [39]. New emerging devices also utilize molecular techniques such as polymerase chain reaction (PCR) to test infectious diseases in sufficiently small devices, enabling real-time data transmission and analysis [40]. Furthermore, technological advances have facilitated the miniaturization and higher portability of POCT devices, allowing for on-the-spot testing and faster diagnosis and treatment decisions [39]. These developments are expanding the applications of POCT devices beyond traditional healthcare settings, broadening access to healthcare and reducing the burden on centralized laboratories [39]. The underlying technology and the range of test parameters available for POCT are evolving rapidly, with innovations in cellphone-based technologies, paper-based assays, lab-on-a-chip platforms, and novel assay formats paving the way for robust, automated, simplified, and cost-effective POCT [41]. Therefore, these emerging technologies are revolutionizing POCT, making it more accessible, accurate, and effective across various healthcare settings.

Wearable and Sensor Technologies

Wearable and sensor technologies are at the forefront of innovative strategies for detecting and monitoring microvascular complications in individuals with T2DM. These technologies present the potential for continuous and non-invasive monitoring of various physiological parameters, offering a valuable tool for early detection and management of complications. Wearable sensors, heralded as promising technologies, have found applications in personalized health monitoring, tracking sports and fitness activities, and medical diagnostics [42]. Notably, these devices have evolved to be thinner, softer, and stretchable, allowing seamless integration with the body through physical, chemical, and biological sensing mechanisms while possessing remote communication capabilities [42].

These sensors have diverse applications, monitoring physiological parameters such as heart rate, blood pressure, glucose levels, and activity levels [43]. Moreover, they hold promise in monitoring the progression of microvascular complications like retinopathy, nephropathy, and neuropathy [44]. The advantages of wearable sensors are manifold, encompassing continuous monitoring, real-time data transmission and analysis, and the capability to detect subtle changes in physiological parameters [44]. Their versatility extends to various settings, including home, work, and clinical environments, positioning them as versatile tools for managing T2DM and its associated complications [42].

However, despite the considerable potential benefits, challenges remain in developing and implementing wearable sensors. Addressing these challenges is crucial and includes the need for accurate and reliable sensors, addressing data privacy and security concerns, and enhancing practical data analysis and interpretation [42]. Wearable and sensor technologies hold promising potential for detecting and monitoring microvascular complications in individuals with T2DM. These emerging techniques can enhance early detection and management of complications, thereby contributing to improved patient outcomes. However, ongoing research and development efforts are necessary to overcome challenges associated with these technologies and ensure their widespread adoption in clinical practice.

Challenges in detection and diagnosis

Variability in Disease Progression

The identification and diagnosis of microvascular complications in T2DM face considerable challenges due to the variability in disease progression. The heterogeneous progression of microvascular complications in T2DM poses a significant obstacle, as it varies substantially among individuals, making developing a universally applicable diagnostic approach challenging [45]. Detection at Subclinical Stages adds another layer of complexity, as many microvascular complications may advance subclinically, lacking overt symptoms until reaching an advanced stage. This characteristic complicates these complications' early detection and diagnosis [46].

The presence of intermittent symptoms in certain complications, such as retinopathy and nephropathy, further compounds the challenge for healthcare providers. The intermittent nature of these symptoms makes accurate diagnosis and management a difficult task [46]. The inconsistency in diagnostic tools introduces additional hurdles, as the effectiveness of these tools can be influenced by factors like age, gender, and other comorbidities. This variability makes it difficult to establish a dependable diagnostic strategy [27]. Limited access to specialized care and diagnostic tools in certain regions exacerbates the challenges faced by healthcare providers in accurately diagnosing and managing microvascular complications in T2DM [27]. Despite these challenges, ongoing research and development in diagnostic technologies, including wearable sensors and point-of-care testing, present promising avenues for detecting and managing microvascular complications in T2DM patients. These emerging techniques can potentially enhance early detection and management, ultimately contributing to improved patient outcomes. However, it is essential to conduct further research and development to address challenges associated with these technologies and ensure their widespread adoption in clinical practice.

Lack of Standardized Diagnostic Criteria

The lack of standardized diagnostic criteria presents a significant challenge in detecting and diagnosing microvascular complications in T2DM. The development of diagnostic criteria for diseases without a gold standard poses significant challenges, and differences in resources and feasibility have limited the development of standardized diagnostic criteria [47]. Diagnostic criteria aim to identify all individuals with the disease, including those with unusual features or presentations, while achieving a relatively homogeneous disease population is essential for any classification criteria [47]. However, to be highly sensitive while preserving acceptable specificity, diagnostic criteria must allow for all the heterogeneous manifestations of the disease, which may vary significantly among individuals [47]. The lack of standardized diagnostic criteria can lead to consistency in diagnostic tools' effectiveness, making it challenging to establish a reliable diagnostic strategy [48]. Also, correct diagnosis remains the main challenge in clinical practice, while correct classification is highly relevant for research [49]. Therefore, it is essential to develop a standardized diagnostic framework that can be adapted to local conditions, taking different pre-test probabilities and the prevalence of 'mimickers' into account [49]. The lack of standardized diagnostic criteria presents a significant challenge in detecting and diagnosing microvascular complications in T2DM. Developing a standardized diagnostic framework adapted to local conditions is essential to improve the early detection and management of complications, ultimately contributing to better patient outcomes.

Patient Adherence to Monitoring Protocols

The challenge of patient adherence to monitoring protocols significantly impedes the detection and diagnosis of microvascular complications in T2DM. Forgetfulness poses a common issue, with patients neglecting to monitor their blood glucose levels or adhering to prescribed monitoring protocols. This results in inconsistent data and compromises the effectiveness of diabetes management [48]. Insufficient education exacerbates the problem, as many patients may not fully understand the importance of self-monitoring and the correct usage of monitoring equipment, leading to errors and inconsistencies in the collected data [48]. Financial barriers further compound the challenges, especially in resource-limited settings, where the high costs associated with monitoring equipment and test strips can hinder access to these crucial technologies [48].

The burden of self-testing proves to be a significant deterrent, as regular monitoring of blood glucose levels can be time-consuming and burdensome for patients, resulting in non-adherence to monitoring protocols [48]. A lack of adequate support adds to the complexity, with some patients lacking access to healthcare professionals or support groups, hindering their ability to adhere to monitoring protocols and effectively manage their diabetes [48]. To overcome these challenges, developing strategies that promote patient adherence to monitoring protocols is imperative. This includes providing comprehensive education, offering financial assistance to mitigate cost barriers, and improving the user-friendliness of monitoring equipment. Additionally, integrating emerging technologies, such as mobile applications and wearable devices, holds promise in enhancing patient adherence to monitoring protocols and improving the overall management of T2DM and its associated complications.

## Conclusions

In conclusion, this comprehensive review of emerging techniques for detecting microvascular complications in T2DM has revealed a dynamic landscape marked by diverse diagnostic modalities. From advanced imaging technologies such as OCT to genomic and proteomic approaches, emerging tools reflect a promising era in precision diagnostics. The potential for personalized medicine has emerged as a pivotal theme, advocating for tailored diagnostic strategies based on individual patient characteristics. However, challenges such as the lack of standardized diagnostic criteria and issues related to patient adherence underscore the need for collaborative efforts. Therefore, a call to action is essential. Enhanced collaboration among clinicians, researchers, industry stakeholders, and policymakers is imperative for developing standardized criteria and translating emerging technologies into routine clinical practice. Increased investment in research, emphasis on patient empowerment through education, and the seamless integration of emerging diagnostic techniques into healthcare systems are crucial steps. Through this concerted effort, we can envision a transformative shift in detecting and managing microvascular complications in T2DM, ultimately leading to improved patient outcomes and a healthier future for those affected by this prevalent metabolic disorder.
